# miR-198 inhibits the progression of renal cell carcinoma by targeting BIRC5

**DOI:** 10.1186/s12935-021-02092-7

**Published:** 2021-07-21

**Authors:** Chao Yuan, Zhenhong Su, Shengjie Liao, Duanzhuo Li, Zhiwen Zhou, Yawen Wang, Mingchun Quan, Lingling Zeng, Cai Lv, Chenyi Shen, Weida Gong, Jianfeng Wu, Xiaogang Chen, Wenbing Hu, Xu Lv, Wenxia Si, Xin Yu

**Affiliations:** 1 Hubei Key Laboratory for Kidney Disease Pathogenesis and Intervention, Hubei Polytechnic University School of Medicine, Xialu District guilin north, road no. 16, Huangshi, 435003 Hubei China; 2 Department of Urology, Haikou Municipal Hospital, Haikou, 570208 Hainan China; 3 Yixing Cancer Hospital, Dongshan Dong Lu No. 45, Yixing, 214200 Jiangsu China; 4 Huangshi Central Hospital, Affiliated Hospital of Hubei Polytechnic University, Huangshi, 435000 Hubei China; 5 Zhaoqing Medical College, Zhaoqing, 526020 Guangdong China

**Keywords:** Renal cell carcinoma, miR-198, *BIRC5*/survivin, Apoptosis

## Abstract

**Background:**

miR-198 is involved in the formation, migration, invasion, and metastasis of various malignant cancers. However, the function and mechanism of action of miR-198 in the tumorigenesis of renal cell carcinoma (RCC) remain elusive. Here, we aimed to explore the role of miR198 in RCC.

**Methods:**

Immunohistochemistry was performed to estimate the level of survivin in RCC sections. Quantitative real-time polymerase chain reaction was performed to determine the expression level of miR-198 in fresh RCC tissues. Furthermore, the target relationship between miR-198 and BIRC5 was predicted using the TargetScanHuman 7.2 database and verified via dual-luciferase reporter assay and western blotting. The effects of miR-198 on the viability, apoptosis, invasion, and migration of A498 and ACHN cells were studied using Cell Counting Kit-8, flow cytometry, transwell migration assay, and wound healing assay, respectively. Additionally, a xenograft nude mouse model was established to evaluate the effect of miR-198 on RCC tumorigenesis.

**Results:**

The expression levels of *BIRC5* and miR-198 were respectively higher and lower in RCC tissues than those in normal adjacent tissues. Furthermore, miR-198 could inhibit luciferase activity and reduce the protein level of survivin without affecting the BIRC5 mRNA levels. miR-198 inhibited cell viability, migration, and invasion and promoted cell apoptosis; co-transfection with *BIRC5* could rescue these effects. Moreover, miR-198 could repress tumor growth in the xenograft nude mouse model of RCC.

**Conclusions:**

Our study demonstrates that miR-198 suppresses RCC progression by targeting *BIRC5*.

**Supplementary Information:**

The online version contains supplementary material available at 10.1186/s12935-021-02092-7.

## Background

Renal cell carcinoma (RCC) is the most invasive and common renal cancer in adults. Globally, the number of RCC cases is increasing annually owing to the presence of multiple etiologies, [1]. Furthermore, only up to 10% of patients with RCC present with characteristic clinical symptoms due to the lack of early warning signs [1]. Approximately 30% of patients with RCC present with metastases at the time of diagnosis [2]. RCC is resistant to radiotherapy and chemotherapy, and early diagnosis and successful surgical resection can save the patient’s life [1]. The 5-year survival rate of patients with stage I, II, III, and IV renal cancer after treatment is 92, 86, 64, and 23%, respectively [3]. These data suggest that the early diagnosis and prediction of renal cancer leads to an increased patient survival rate. In recent years, advanced drugs for malignant RCC that interfere with molecular targets involved in tumor growth and development have been used in clinical practice[2–4]. The developed drugs, including everolimus, sunitinib, and axitinib have achieved remarkable results but have not cured patients [4–6]. Therefore, it is crucial to identify biomarkers of RCC and explore their molecular mechanisms to design new therapy targets for RCC.

MicroRNAs (miRNAs) are small non-coding RNAs that are responsible for the post-transcriptional regulation of gene expression by targeting their corresponding mRNA. The abnormal expression of miRNA has been correlated to various diseases, including cancers [7]. Dysregulated miRNAs are considered novel tumor inhibitors or oncogenes vital for the development and progression of cancer [8]. miR-198 has reportedly played an essential role in many cancers, including lung, breast, gastric, and liver cancers [9–12]. To illustrate, Hu et al. revealed that miR-198 acted as a tumor suppressor in aggressive breast cancer by not only inhibiting cell proliferation and migration but also promoting cell adhesion [11]. However, Liang et al. reported that miR-198 induced oncogenesis of lung cancer via upregulating livin expression [13]. However, the role of miR-198 in renal tumors has not been currently elucidated.

*BIRC5* gene, the whole neme is Baculoviral inhibitor of apoptosis repeat-containing 5, which encodes the Survivin protein, i.e., the smallest member of the inhibitor of apoptosis family. *BIRC5* is involved in many cancers, and the survivin protein has been identified as a cancer biomarker [14–16]. *BIRC5* regulates cancer development by inhibiting cell apoptosis and inducing cell proliferation [17]. Additionally, increasing evidence demonstrates that BIRC5 is influences the aggressiveness of clear-cell RCC, and a high survivin expression level predicts a poor patient outcomes [18].

In this study, we aimed to explore the role of miR-198 in renal carcinoma and reveal its underlying molecular mechanism.

## Methods

### Clinical specimens

Thirty sets of kidney specimens, including renal carcinoma tissues and adjacent normal tissues, were collected from patients who underwent surgical resection of tumors at Haikou Municipal Hospital affiliated to Xiangya School of Medicine of Central South University from June 2016 to June 2018. This study was approved by the Ethics Committee of Hubei Polytechnic University and Haikou Municipal Hospital. All patients provided written informed consent.

### Immunohistochemistry

RCC tissues and adjacent tissues were stained with an antibody against survivin (10,508–1-AP, Proteintech), secondary antibody (GB23303; Servicebio), and diaminobenzidine reagent (DAB Horseradish Peroxidase Color Development Kit; Beyotime) following a previously described method [19]. The stained tissues were imaged using an Olympus microscope at × 200 and × 400.

### Cell culture

The human RCC cell line A498 and ACHN (China Center for Type Culture Collection, Wuhan, China) were cultured in Dulbecco’s modified Eagle’s medium (DMEM; Hyclone) containing 10% fetal bovine serum (FBS; Hyclone) in a cell incubator (Thermo) with 5% CO_2_ at 37 °C. Cell transfection was performed with Lipofectamine™ 2000 reagent (Invitrogen) according to the manufacturer’s instructions.

### Plasmids

The 600-bp 3′-UTR (untranslated region) of *BIRC5*, which encodes survivin, was amplified by polymerase chain reaction (PCR) using human genomic DNA as the template. The PCR product was digested with *Sac*I and *Hind*III and cloned into the pMIR-REPORT™ luciferase vector (Promega), resulting in a plasmid encoding a luciferase reporter protein BIRC5-WT. The miR-198 binding site in the 3′-UTR of the gene encoding BIRC5-WT was mutated using point mutation by PCR as previously described [20] so that the mutant gene encodes reporter BIRC5-MUT. The *BIRC*5 eukaryotic expression plasmid FLAG-BIRC5 was cloned from cDNA and inserted into pCDNA3.1-FLAG (Addgen) with *Kpn*I and *Hind*III restriction sites. The primers used are listed in Table 1. miR-198 mimic, miR-198 inhibitor, and scrambled miRNA mimic were synthesized by RioboBiO (Guang Zhou, China).

**Table 1:** The primers for plasmids

Name	Forward primer	Reverse primer
FLAG-BIRC5	cccAAGCTTATGGGTGCCCCGACGTTGC	ggGGTACCTCAATCCATGGCAGCCAGCTG
BIRC5-WT	gcgcgcGAGCTCGGCCTCTGGCCGGA	gcgcgcAAGCTTAAGCCATGTTGTTAA
BIRC5-MUT	CAGTGAATGTGTTTAAACCTC	AACAACATGAGGTTTAAACACAT

### Extraction of total RNA and quantitative real-time PCR

Total RNA was extracted from the clinical samples and cell lines using TRIzol reagent (TaKaRa). Complementary DNA (CDNA) of *BIRC5* was reverse transcribed from RNA using HiScript II Q RT SuperMix (R223-01, Vazyme). mRNA expression was determined by quantitative reverse transcription PCR (QRT-PCR) with ChamQ™ SYBR® qPCR Master Mix (Q311-02, Vazyme) using QuantStudio Q5 (Applied Biosystems). GAPDH was used as the internal reference for BIRC5 mRNA. The relative expression of the genes was calculated using the 2^−ΔΔCt^ method. The following primers were used for quantitative PCR:

**Table:** 

GAPDH-F:	5’ATCGTGGAAGGACTCATGACC3’
GAPDH-R:	5’AGGGATGATGTTCTGGAGAGC3’
BIRC5-F:	5’AGGACCACCGCATCTCTACAT3’
BIRC5-R:	5’AAGTCTGGCTCGTTCTCAGTG3’

miR-198 quantification was performed using the following protocol: CDNA of miR-198 and U6 were reverse transcribed using miRNA 1st Strand cDNA Synthesis Kit (MR101-01, Vazyme) with miR-198 RT primer (ssD809230220, RioboBiO) and U6 RT primer (ssD0904071008, RioboBiO), respectively. Then we estimated the miR-198 expression level in the miR-198 CDNA library using miRNA Universal SYBR qPCR Master Mix (MQ101-01) with miR-198 forward (ssD809230912, RioboBiO) and reverse (ssD089261711, RioboBiO) primers. Furthermore, we tested the U6 expression level in the U6 cDNA library with U6 forward (ssD0904071006, RioboBiO) and reverse (ssD0904071007, RioboBiO) primers.. The data were normalized to the U6 expression level and analyzed using the 2^−ΔΔ Ct^ method (Additional file 2).

### Western blots

A498 and ACHN cells were transfected with the indicated miR-198 or scrambled miRNA mimic for 48 h. Total protein was then extracted from the cells using radioimmunoprecipitation assay (RIPA) lysis buffer (Beyotime) according to the manufacturer’s protocol. Next, the cell lysates were subjected to western blotting with antibodies against survivin (10508-1-AP, Proteintech), β-tubulin (HRP-66031, Proteintech) or β-Actin (GB11001, Servicebio) (Additional file 2).

Each group of tumor tissues in the mouse model was assessed for survivin expression; 20 mg of tumor tissues were weighed and quick-frozen in liquid nitrogen, followed by homogenization with 500 μl RIPA lysis buffer. After homogenization, the lysates were incubated in a 4 °C shaking table for 2 h to allow further lysis; the lysates were then centrifuged at 13000 g × 10 min, 4 °C. The supernatant was mixed with loading buffer and boiled at 100 °C for 15 min, then stored at − 80 °C.

### Dual-luciferase reported assay

Luciferase assays were performed as described previously [20]. Briefly, A498 and ACHN cells cells were cultured in a 24-well plate and co-transfected with 150 ng of *BIRC5-WT* or *BIRC5-MUT* plasmid with 100 nM miR-198, scrambled mimic of miR-198, or 10 ng of pRL-TK vector (Promega). After 48 h, the cells were lysed, and the lysates were used for luciferase assays using the Dual-Glo Luciferase Assay kit (Promega). Fluorescence was measured using the Promega GloMaxTM 20/20 Luminometer, and luciferase activity was normalized with the ratio of fluorescence values of firefly luciferase and *Renilla* luciferase.

### Cell viability assay

A498 and ACHN cells were seeded in a 96-well plate and transfected with a scrambled mimic of miR-198, miR-198, and miR-198 + FLAG-BIRC5 for 0, 24, and 48 h. Then, cell viability was determined using a Cell Counting Kit-8 Kit (Dojindo Laboratories, Japan) according to the manufacturer’s instructions. Cell viability was determined by measuring the absorbance at 450 nm using a microplate reader. We regarded the absorbance at 450 nm as the cell viability rate.

### Cell cycle assay

The effect of miR-198 on the cell cycle in RCC was assayed using the Cell Cycle Staining Kit (Beyotime). After transfection with miR-198 or its scrambled mimic for 48 h, A498 cells were washed with phosphate-buffered saline (PBS), trypsinized, fixed in 70% ice-cold ethanol, and incubated with 100 mg/mL RNase and 4 mg/mL propidium iodide (PI) in PBS. The cell cycle phase of the cells was analyzed using a flow cytometer (FC500; Beckman Coulter) and was analyzed using CXP software (Beckman Coulter).

### Apoptosis assay

The effects of miR-198 on cell apoptosis were measured using the Annexin V-FITC/PI apoptosis detection kit (Beyotime) according to the manufacturer’s instructions. A498 and ACHN cells cells were transfected with a scrambled mimic of miR-198, miR-198, and miR-198 + FLAG-BIRC5 for 48 h and then treated with puromycin aminonucleoside (100 µg/mL) for 48 h to induce apoptosis. The cells were then washed twice with cold PBS, digested with trypsin with EDTA, neutralized with DMEM, and harvested. After washing with cold PBS two times, the cells were then resuspended in 200 µL of binding buffer and stained with 5 µL of Annexin V-FITC and 10 µL of PI at room temperature for 15 min. Cell apoptosis was analyzed using a Coulter FC500 instrument. The flow cytometry data were analyzed using CXP software. We calculated the percentage of apoptotic cells by the percentage of cells in the upper right quadrant (Annexin V-FITC-positive, PI-positive) and cells in the lower right quadrant (Annexin V-FITC positive, PI-negative).

### Wound healing assay

A498 and ACHN cells were transfected with a scrambled mimic of miR-198, miR-198, and miR-198 + FLAG-BIRC5. After 36 h, the cells in the middle of the well were scratched using a 10 μL pipette tip. The cells were visualized under × 100 magnification (Olympus microscope) at 0 and 24 h after scratching, and the extent of cell migration was analyzed using ImageJ software.

### Transwell assay

A498 and ACHN cells were transfected with a scrambled mimic of miR-198, miR-198, and miR-198 + FLAG-BIRC5 for 48 h. Next, the cells were digested, resuspended, and plated in a transwell chamber (Corning) previously coated with Matrigel 1:8 diluted with serum-free DMEM (BD), with 100 µL of serum-free DMEM, while out the part with 500 µL of 10% FBS DMEM. The cells in the transwell chamber were cultured for 12 h in an incubator, fixed with methyl alcohol for 20 min, stained with 0.1% crystal violet for 10 min, washed several times with 1 × PBS, and imaged at 40 × using an Olympus light microscope. The number of invasive cells was counted using ImageJ software.

### Xenograft tumor model

The mouse studies were approved by the Ethics Committee of Hubei Polytechnic University. Male BALB/C-nu/nu nude mice aged 6–8 weeks were used in the experiments. A498 cells were transfected with miR-198, its specific inhibitor, or its scrambled mimic for 48 h. The cells were then harvested, washed with cold PBS, and suspended at a density of 5 × 10^6^ cells/mL in PBS. The well-treated A498 cells, at a density of 5 × 10^5^ cells/100 μL, were injected subcutaneously in the lateral side of the right rear leg (n = 6). Tumor diameters were measured using vernier calipers every 2 days, and tumor volumes were calculated using the formula (width^2^ × length/2). The mice were sacrificed at the end of each experiment, and tumors were excised, weighed, imaged, and fixed for further characterization.

### Statistical analysis

Orange 8.5 and GraphPad Prism 6.0 software were used for statistical analyses. All data were presented as means ± standard deviations. A t-test and one-way ANOVA (analysis of variance) were used to compare differences between groups. *P* < 0.05 was considered statistically significant.

## Results

### Expression of *BIRC5* in RCC

**Fig. 1:**
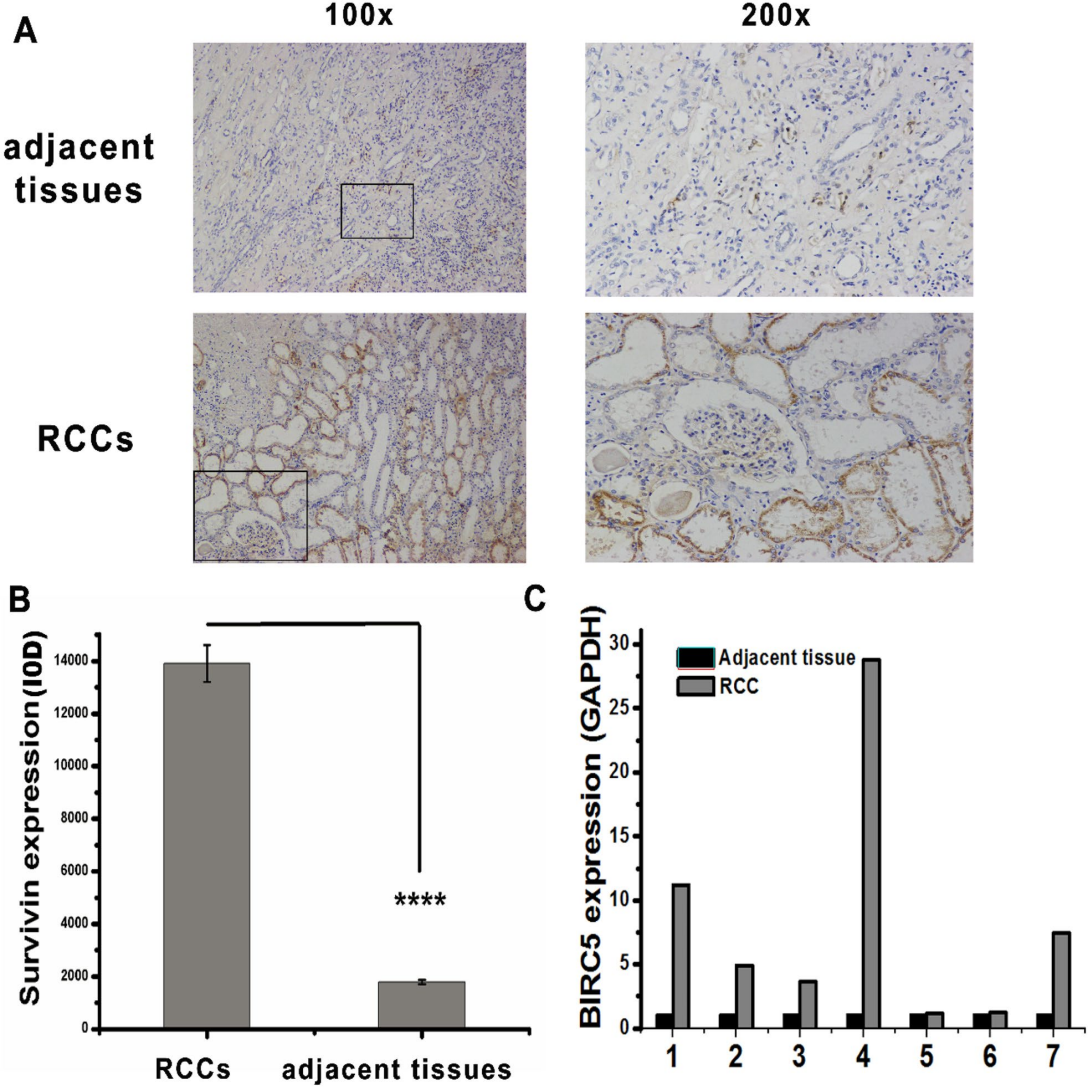
*BIRC5* is overexpressed in renal cell carcinoma. **A** Fifteen pairs of renal cell carcinoma (RCC) tissues and adjacent tissues were subjected to immunohistochemistry staining to characterize the level of survivin. **B** Statistical analysis. Analysis of the positive signal (IOD) of survivin in RCC and adjacent tissues using image pro plus 6.0. ****P < 0.0001. **C** qRT-PCR was performed to compare the mRNA expression of *BIRC5* in RCC and adjacent tissues. The expression of *BIRC5* was significantly upregulated in RCC tissues

We investigated the role of *BIRC5* in RCC by characterizing its expression using quantitative PCR and immunohistochemistry (IHC). *BIRC5* was overexpressed in 7 RCC patients compared with that in adjacent tissues (Fig. 1C). The level of survivin was assayed using IHC with the survivin antibody, and RCC tissues exhibited intense survivin staining (Fig. 1A–B). Surprisingly, Zhang et al. also reported a high expression of *BIRC5* in RCC tissues and RCC cell lines, especially in A498 cells [21]. These results suggest that *BIRC5* contributes to RCC.

### miR-198 suppresses *BIRC5* expression

**Fig. 2:**
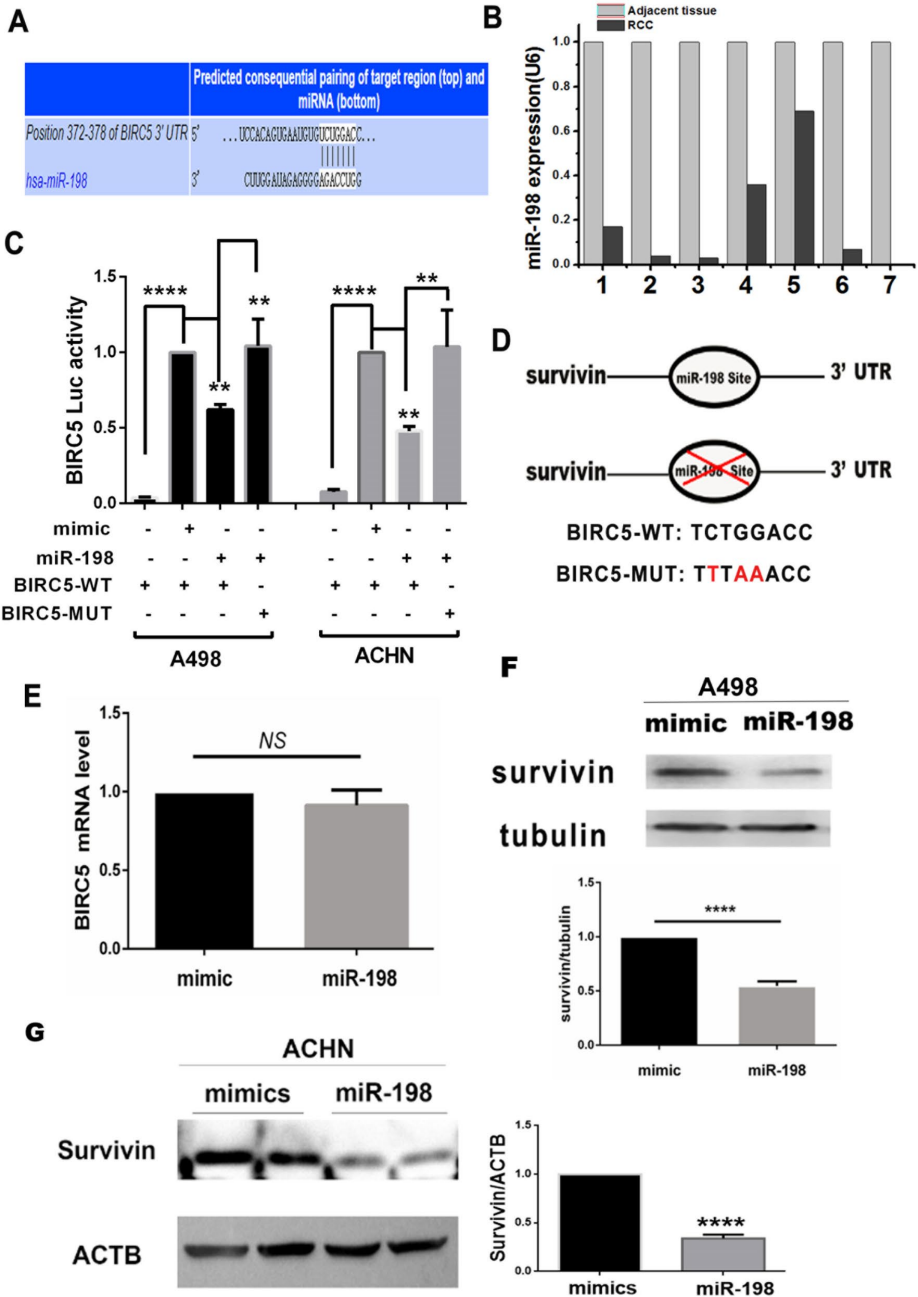
miR-198 decreases the expression of *BIRC5*. **A** Bioinformatics analysis of miR-198 and *BIRC5* using TargetScanHuman 7.2. The graphic model of the binding site of miR-198 in the 3′-UTR of *BIRC5*. **B** qRT-PCR. miR-198 expression was quantified in 7 pairs of fresh RCC and adjacent tissues. **C** Luciferase assay. A498 and ACHN cells were co-transfected with *BIRC5-WT* + pRL-TK, mimic + *BIRC5-WT* + pRL-TK, miR-198 + *BIRC5-WT* + pRL-TK, and miR-198 + *BIRC5-MUT* + pRL-TK; The cells were harvested 48 h later for dual-luciferase assay. **D** Point mutation model of *BIRC5* dual-luciferase reporter vector. *BIRC5-WT* is the luciferase reporter vector with wild-type *BIRC5* 3′-UTR, and *BIRC5-MUT* is the luciferase reporter vector with *BIRC5* 3′-UTR with a mutated miR-198 binding site. **E** A498 cells were treated with the scrambled mimic and miR-198; After 48 h, qRT-PCR was performed. **P < 0.01; ****P < 0.0001. **F** A498 cells were treated as described in (**E**); after 48 h, the cell lysates were subjected to western blotting with survivin and tubulin antibodies. ****P < 0.0001. **G** ACHN cells were treated with the scrambled mimic and miR-198; after 48 h, the cells underwent western blotting analysis with indicated antibodies. ****P < 0.0001

To get predicted targets of miR-198, Huang et al. conducted the prediction process online via 14 miRNA database and selected 127 mRNAs as the predicted targets [22]. *BIRC5* was of particular interest since its expression has been identified as a cancer biomarker in many types of cancers [14–16]. Using TargetScan Human 7.2 database, we located one binding sites for miR-198 at the 3 ‘UTR of the BIRC5 mRNA, however the miR-198 site within BIRC5 3'UTR is not conserved (Fig. 2A). To verify weather the miR-198 binds to *BIRC5* 3'UTR, we cloned the wild-type and mutated 3′-UTR of BIRC5 into a pMIR-reporter vector as BIRC5-WT and BIRC5-MUT, respectively, and performed dual-luciferase reporter assay in A498 and ACHN cells. The luciferase activity of the cells co-transfected with miR-198 and *BIRC5-WT* was significantly lower than that of the cells co-transfected with the scrambled mimic and *BIRC5-WT*; in contrast, the co-transfection of miR-198 and *BIRC5-MUT* did not affect the luciferase activity (Fig. 2C–D). These results indicate that miR-198 could bind to the 3′-UTR of *BIRC5* in RCC. We also examined the expression of miR-198 in eight pairs of fresh RCC tissues and adjacent tissues using quantitative PCR. The expression of miR-198 in RCC tissues was significantly lower than that in adjacent tissues (Fig. 2B). Furthermore we porfermed the QRT-PCR and Western blot to assessed the effect of miR-198 on *BIRC5* expression, and found that miR-198 did not affect the mRNA expression of *BIRC5* (Fig. 2E) but decreased the level of survivin (Fig. 2F–G). These results show that miR-198 is bound to the 3′-UTR of *BIRC5* and suppressed the level of survivin.

### miR-198 promotes the apoptosis of RCC cells and inhbits the cell viability

**Fig. 3:**
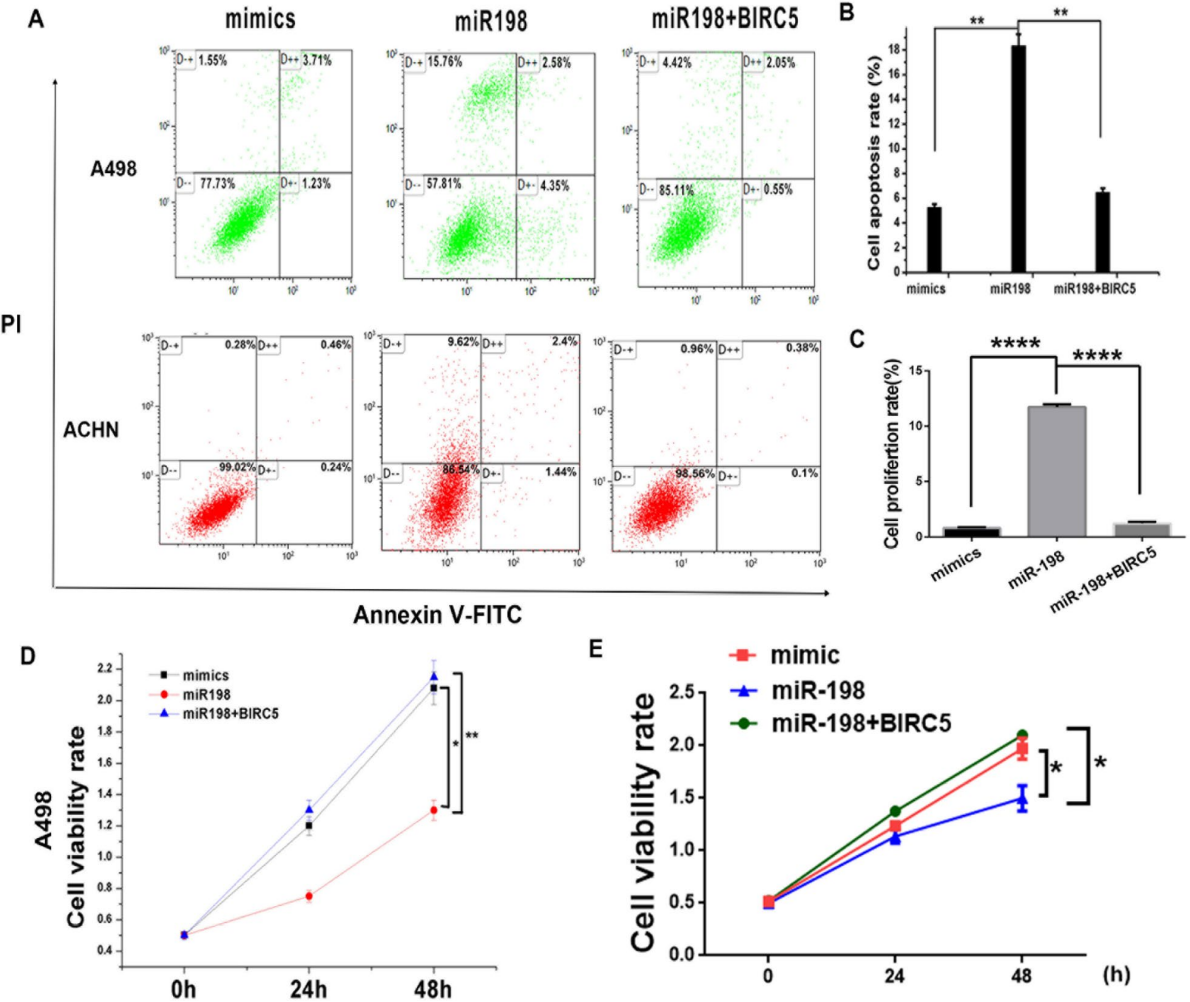
miR-198 affects cell growth. **A** Apoptosis assay. A498 and ACHN cells were transfected with the scrambled mimic, miR-198, and miR-198 + FLAG-BIRC5 for 36 h, following which, they were treated with 100 μg/ml of puromycin for 36 h, and analyzed using flow cytometry. **B** Statistical analysis of the apoptosis rate in A498 cells in (**A**). **P < 0.01. **C** Statistical analysis of the apoptosis rate in ACHN cells in (**A**). ****P < 0.0001. **D** CCK8 assay of A498 cells. A498 cells were transfected with the scrambled mimic, wild-type miR-198 and miR-198 + FLAG-BIRC5. Then, 5 μL of CCK-8 solution was added to the cells, and cell viability rate was determined using an enzyme marker at 0, 24, and 48 h. *, *P* < 0.05; **P < 0.01. **E** CCK8 assay of ACHN cells. ACHN cells were treated as described in (**D**). *P < 0.05

*BIRC5* reportedly plays a crucial role in RCC development, including in cell viability, metastasis, and survival [21]. Our previous results confirmed that miR-198 can decrease the protein expression level of *BIRC5* (Fig. 2). Thus, we studied the effects of miR-198 on RCC development by transfecting the A498 and ACHN cells with scrambled mimic, miR-198, and miR-198 + FLAG-BIRC5 and measuring cell viability, apoptosis, and cell cycle experiments. We examined the viability of A498 and ACHN cells in 96-well plates at 0, 24, and 48 h three times and found that miR-198 suppressed the viability of RCC cells and *BIRC5* blocked the effect of miR198 (Fig. 3D–E). Additionally, apoptosis assays showed that miR-198 increased the apoptosis of RCC cells, whereas co-transfection with BIRC5 could rescue the apoptosis (Fig. 3A–C). Furthermore, we performed the cell cycle assay and found that miR-198 did not affect the cell cycle (Additional file 1: Fig. S1). These results reveal that miR-198 might impede cancer cell development through apoptosis by suppressing *BIRC5* expression.

### miR-198 decreases the migration and invasion capabilities of RCC

**Fig. 4:**
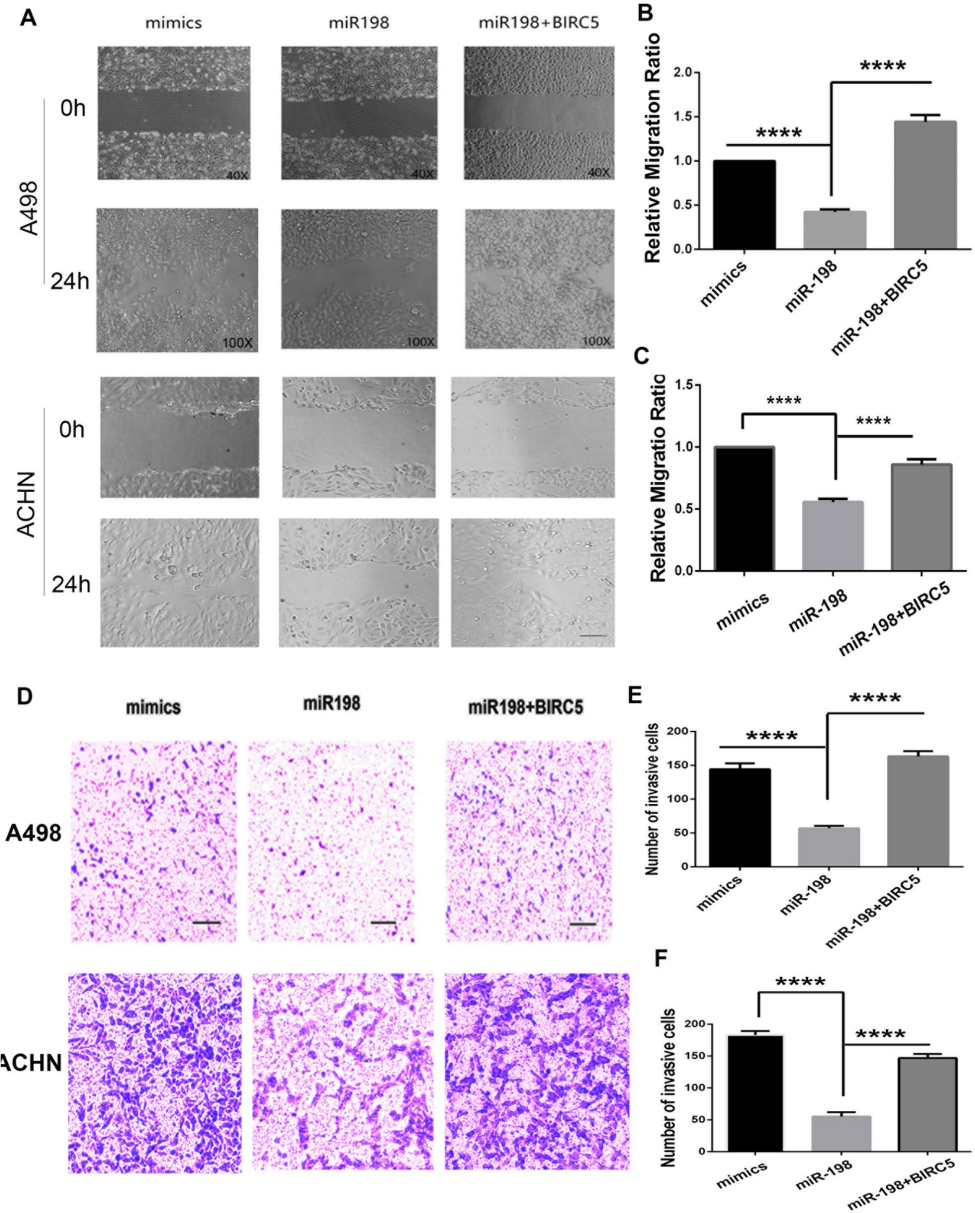
miR-198 regulates cell migration and invasion. **A** A498 and ACHN cells were transfected with the scrambled mimic of miR-198, wild-type miR-198, and miR-198 + FLAG-BIRC5; after 36 h, cells were analyzed using the wound healing assay, and the results were determined at 0 and 24 h. **B** Statistical analysis of the cell migration ratio of A498 cells using Image J. ****P < 0.0001. **C** Statistical analysis of the cell migration ration of ACHN cells. ****P < 0.0001. **D** Transwell assay. A498 and ACHN cells were treated as described in (**A**), after 36 h, the cells were digested, resuspended, and cultured in a Matrigel-coated transwell chamber and tested for invasion after 12 h. **E** Statistical analysis of cell invasion of A498 cells using Image J. ****P < 0.0001. **F** Statistical analysis of cell invasion of ACHN cells. ****P < 0.0001

Understanding how tumor cells develop heterogeneity, invade local tissues, and spread to distant tissues is a primary goal of cancer research [23]. Therefore, we studied the role of miR-198 in RCC progression using wound healing assay and transwell assay. A498 and ACHN cells were transfected with scrambled mimics of miR-198, miR-198, and miR-198 + FLAG-BIRC5. After 36 h, the cells were subjected to the wound healing and transwell assays. The wound healing assay results showed that, compared with the control group, miR-198 overexpression inhibited the migration of RCC cells in miR-198 group, and BIRC5 co-expression with miR-198 reversed this effect (Fig. 4A–C). Similarly, the transwell assay results showed that miR-198 overexpression significantly decreased the invasion ability of RCC cells and that BIRC5 suppressed this effect of miR-198 (Fig. 4D–F). These results suggest that miR-198 suppressed cancer cell development by inhibiting cancer cell migration and invasion.

### miR-198 inhibits RCC growth in vivo

**Fig. 5:**
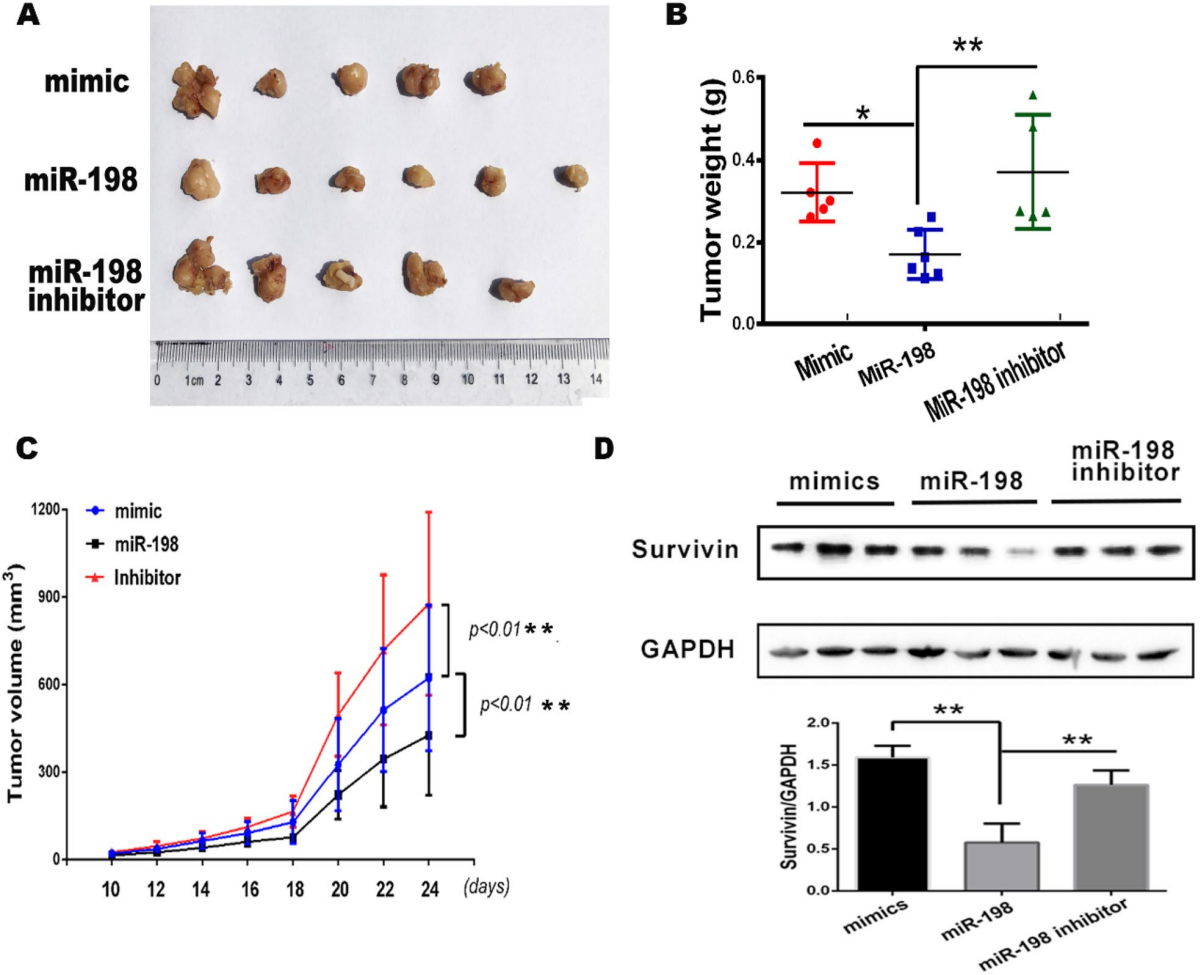
miR-198 inhibits the growth of renal cell carcinoma. A498 cells were transfected with the scrambled mimic of miR-198, wild-type miR-198, or miR-198 inhibitor. After 36 h, the cells were collected and injected into nude mice (n = 6). Each tumor’s width and length were measured daily; after 24 days, the nude mice were sacrificed to excise the tumor. **A** Tumor images. **B** Weight of the tumors. *P < 0.05; **P < 0.01. **C** Growth curve of the tumors. **, *P* < 0.01. **D** The expression of Survivin in each group of tumor tissues (n = 3) was tested by Western Blot with indicated antibodies. **P < 0.01

The results of the in vitro experiments suggested that miR-198 inhibited RCC via inhibiting cell viability, migration and invasion and promoting apoptosis (Figs. 3 and 4). Then, we evaluated whether miR-198 affected renal tumor growth in vivo using a xenograft tumor model. We transfected A498 cells with miR-198, miR-198 scrambled mimic, or miR-198 inhibitor. We injected the transfected cells into nude mice, monitored tumor growth by measuring tumor length and width every 2 days, and excised the tumor after 22 days. Compared with the scrambled mimic, miR-198 could suppress the renal tumor size, whereas the miR-198 inhibitor could block the repressive effect of miR-198 on renal tumor size (Fig. 5A). The growth curve of a renal tumor showed that miR-198 could slow the tumor growth rate, and its effect was blocked by miR-198 inhibitor (Fig. 5C). In addition, we observed a similar result on the effect of miR-198 and its variants on tumor weight (Fig. 5B). Furthermore, we investigated the expression of survivin in each group of tumor tissues through western blotting and found that, compared with the mimic group, the expression of survivin was downregulated in the miR-198 group, and the miR-198 inhibitor could block its downregulation (Fig. 5D). Data from the in vivo tumor model suggest that miR-198 suppresses renal tumor growth.

## Discussion

In this study, the expression level of miR-198 was downregulated in RCC tissues compared with that in adjacent tissues in all 7 pairs of RCC samples (Fig. 2B). Moreover, the expression level of *BIRC5* in RCC tissues was higher than that in adjacent tissues (Fig. 1). Owing to limitations in sample size, further studies with larger sample sizes to confirm that miR-198 is a potential marker of RCC are warranted.

miR-198 overexpression could significantly reduce cell viability and promote cell apoptosis by targeting *BIRC5* (Fig. 3); furthermore, it can decrease cancer cell migration and invasion, whereas *BIRC5* coexpression could reverse this effect (Fig. 4). The in vivo tumor growth model revealed that miR-198 could reduce the renal tumor size, weight, and growth rate; the tumor growth rate in the miR-198 inhibitor group was similar to that in the mimic group (Fig. 5). Therefore, it is highly likely that the low expression level of miR-198 is responsible for RCC development and tumor growth. Mechanistically, we showed that miR-198 inhibited renal cell growth by posttranslationally suppressing survivin levels by binding to the 3′-UTR of *BRIC5* and reducing protein translation without affecting mRNA transcription (Fig. 2). Collectively, we conclude that *BIRC5*/survivin is a downstream target of miR-198*.* Similar to this finding, the upregulation of *BIRC5* expression in RCC tissues and the promotion of tumorigenesis were reported by Zhang et al. [21]. More interestingly, Huang et al. showed that miR-198 as well as its potential target genes, including *BIRC5*, were downregulated in HCC [22]. These reports are all consistent with our results.

A previous study reported that low miR-198 expression in other types of tumors and miR-198 overexpression could inhibit tumorigenesis. For instance, miR-198 inhibits lung cancer cells and human osteosarcoma by directly targeting FGFR1 and ROCK1, respectively [24]. miR-198 inhibits via the targeting of [25]. However, Liang et al. showed that miR-198 contributed to the oncogenesis of lung adenocarcinoma by inducing livin expression but did not test the expression of miR-198 [13]. Furthermore, miR-198 inhibits HCC cells by targeting the HGF/c-MET pathway [9]. Additionally, miR-198 represses tumor growth and metastasis in colorectal cancer by targeting fucosyl transferase 8 [26]. These studies indicate that miR-198 plays a crucial role in tumorigenesis in various cancer types by targeting different downstream genes.

## Conclusions

In summary, we demonstrated that miR-198 expression is repressed in RCC tissues. The overexpression of miR-198 inhibits A498 cell viability and migration, promotes cell apoptosis, and blocks tumor growth in xenograft nude mouse models. *BIRC5* is a novel target in miR-198-mediated invasion and migration of RCC. We suggest that miR-198 is a potential biomarker and novel therapeutic target for RCC.

## Supplementary Information

**Additional file 1**: **Figure S1**. miR-198 does not affect the cell cycle. (A,B) A498 and ACHNcells were transfected with scrambled mimic of miR-198 and wild-type miR-198. After 36 h, the cells were analyzed using flow cytometry to assess their cell cycle. NS = no significant. (C,D) ACHNcells were treated like (A). NS = no significant.

**Additional file 2**. The original data of RT-PCR and Western Blot.

## Data Availability

All data generated or analyzed during this study are included in this article.

## Abbreviations

RCC: Renal cell carcinoma
BIRC5: Baculoviral inhibitor of apoptosis repeat-containing 5
IHC: Immunohistochemistry
HCC: Hepatocellular carcinoma
PCR: Polymerase chain reaction
TKI: Tyrosine kinase inhibitors
mTOR: Mammalian target of rapamycin
VEGF: Vascular endothelial growth factor
