# Supplementary figures and images for: miR-198 inhibits the progression of renal cell carcinoma by targeting BIRC5
Source: Cancer Cell Int. 2021 Jul 21;21:390. doi: 10.1186/s12935-021-02092-7 (PMC8296723; doi:10.1186/s12935-021-02092-7)

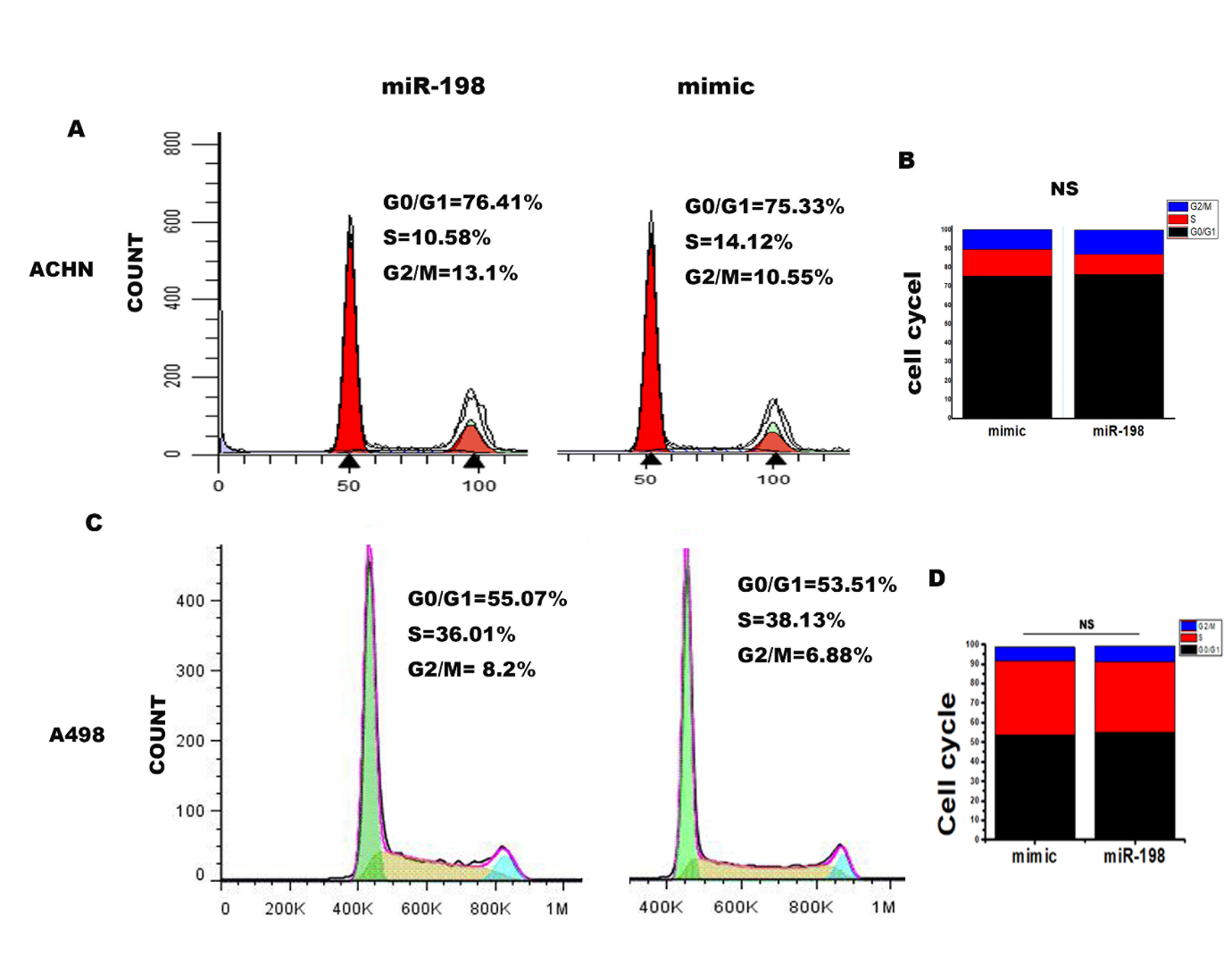


Figure S1

Supplement: Supplementary file 1 — Additional file 1: Figure S1. miR-198 does not affect the cell cycle. (A,B) A498 and ACHNcells were transfected with scrambled mimic of miR-198 and wild-type miR-198. After 36 h, the cells were analyzed using flow cytometry to assess their cell cycle. NS = no significant. (C,D) ACHNcells were treated like (A). NS = no significant. [file 12935_2021_2092_MOESM1_ESM.docx]
