# Supplementary material for: miR-198 inhibits the progression of renal cell carcinoma by targeting BIRC5
Source: Cancer Cell Int. 2021 Jul 21;21:390. doi: 10.1186/s12935-021-02092-7 (PMC8296723; doi:10.1186/s12935-021-02092-7)

**1. qPCR original CT data**

|  |  | **miR-198**  **Ct mean** | **U6**  **Ct mean** |  | **BIRC5**  **Ct mean** | **GAPDH**  **Ct mean** |
| --- | --- | --- | --- | --- | --- | --- |
| 1号病人 | cancer | 34.93 | 21.7 |  | 22.6 | 22.79 |
|  | adj | 36.75 | 26.09 |  | 30.39 | 27.09 |
|  |  |  |  |  |  |  |
| 2号病人 | cancer | 35.13 | 21.23 |  | 20.98 | 20.43 |
|  | adj | 35.07 | 25.81 |  | 27.86 | 25.01 |
|  |  |  |  |  |  |  |
| 3号病人 | cancer | 36.19 | 20.52 |  | 27.75 | 21.8 |
|  | adj | 31.57 | 24.4 |  | 33.52 | 25.7 |
|  |  |  |  |  |  |  |
| 4号病人 | cancer | 30.72 | 20.35 |  | 26.36 | 23.22 |
|  | adj | 30.02 | 21.13 |  | 31.99 | 24 |
|  |  |  |  |  |  |  |
| 5号病人 | cancer | 27.31 | 20.21 |  | 31.83 | 22.35 |
|  | adj | 26.88 | 20.31 |  | 32.98 | 22.92 |
|  |  |  |  |  |  |  |
| 6号病人 | cancer | 34.2 | 24.69 |  | 24.52 | 20.61 |
|  | adj | 31.66 | 25.95 |  | 24.92 | 20.71 |
|  |  |  |  |  |  |  |
| 7号病人 | cancer | 30.64 | 17.25 |  | 30.11 | 28.49 |
|  | adj | 25.49 | 23.67 |  | 34.27 | 29.75 |

**1.1 Original Ct mean of RCC samples for figure 1C and figure 2B**

| **1.2 Original Ct mean of A498 cell line transfected with miR-198 for figure 2f**   \|  \| **BIRC5**  **Ct mean** \| **GAPDH**  **Ct mean** \| \| --- \| --- \| --- \| \| mimic \| 27.368 \| 18.71 \| \| miR-198 \| 27.054 \| 18.59 \| |  |  |
| --- | --- | --- | --- | --- | --- | --- | --- | --- | --- | --- | --- |
|  |  |  |

**2. Western blot original figure for figure 2E**


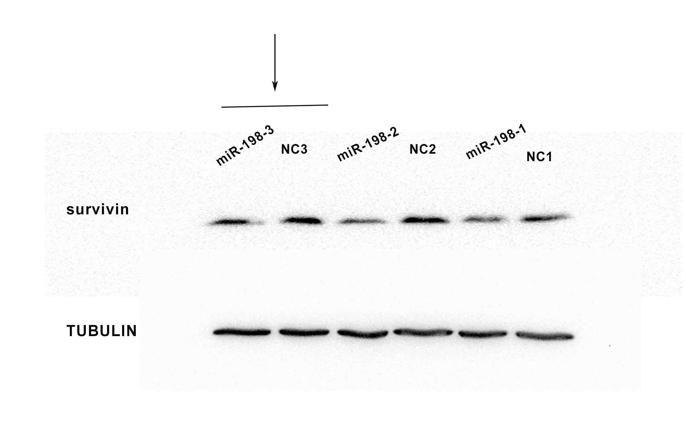


**3. Western blot original figure for figure 2G**


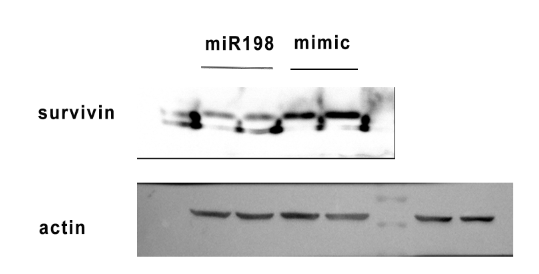


**4.Western blot original figure for figure 5D**


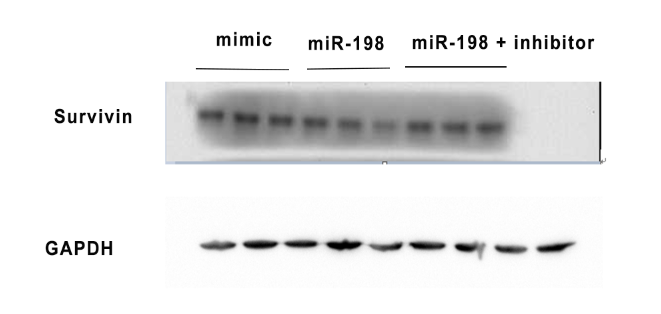

Supplement: Supplementary file 2 — Additional file 2. The original data of RT-PCR and Western Blot. [file 12935_2021_2092_MOESM2_ESM.docx]
